# RNTCP and tuberculosis control - High time to act

**DOI:** 10.4103/0970-2113.68303

**Published:** 2010

**Authors:** Nirmal Kumar Jain

**Affiliations:** *Department of Chest Diseases and TB, SMS Medical College, Jaipur, India. E-mail: jainnkdr@yahoo.co.in*

Revised National Tuberculosis Control Program (RNTCP), based on the Directly Observed Therapy Short-course (DOTS) strategy, began as a pilot project in 1993 and was launched as a national program in 1997 and the entire country was covered under DOTS by 24th March 2006.[1] The program has consistently maintained the treatment success rate of more than 85% and new smear positive (NSP) case detection rate (CDR) of 70%. The DOTS strategy has been able to standardize drug regimens, prevent misuse of drugs and avoid emergence of drug resistance.[2] To achieve this, RNTCP has created a strong and vast infrastructure under it.[1]

However, recent evidences highlight the limitations that bind RNTCP and these might make control of tuberculosis a difficult task. An article by Jain *et al*. in this issue of journal[3] shows the efficacy of DOTS Category-I and Category-III in lymph node tuberculosis to be around 71%. Similar concerns are there about the efficacy of six-month regimen in TB-meningitis and TB of bones and joints since the implementation of DOTS with most experts recommending the duration of therapy to be around 9-12 months.[4,5] The DOTS strategy and its regimens in extra-pulmonary tuberculosis have been the most discussed topics in the core committee’s of medical colleges.

Another article by Ramachandran *et al*. in this issue shows that even after a decade of its implementation, still there is lack of awareness among the patients about the availability and quality of free diagnostic and treatment facilities locally under RNTCP. These patients either avail facilities from hospitals, medical colleges or private practitioners.[6] Considering the current incidence i.e. 1.9 million per year, an average of 23 million new cases of TB have occurred in last 12 years since the country-wide implementation of RNTCP in India. RNTCP has initiated nearly 10 million cases on treatment.[1] Hence, more than half of the total TB patients by pass the local RNTCP services and are either treated in private sectors or are untreated.[7] Of the 8 million doctors in India, about 6 million are engaged in private practice and only 19,000 private practitioners (0.31%) are implementing RNTCP.[1] Still most of the private physicians have practically no access to information or training programs, which accounts for surprising disparity in their management strategies. The program should ensure active involvement of the private sector in case detection and notification and provide them with the standard guidelines of TB care. Health system strengthening by clearing up of the shortage of staff and creating parallel staff for the private sector is needed. A parallel tuberculosis unit (TU) for 5 lakh population as for the government sector with all key staff can be worked out for private sector to ensure good participation as well as efficient monitoring. Medical colleges should be actively involved with activities such as training of senior health professionals and other staffs, delivery of services of RNTCP and operational research with involvement of professors of various departments especially those of orthopedics, neurology and gynecology. The procrastination regarding research in TB should end and there should be active attempts to decentralize the decisions over research in TB at medical college level.

Incidence of mono-drug resistance among new cases in India for INH is 3-32%.[8,9] WHO recommends that in populations with known or suspected high levels of isoniazid resistance, new TB patients may receive HRE as therapy in the continuation phase as an acceptable alternative to HR.[10] Evidence shows that even adequate implementation of a National Tuberculosis Control Program can lead to selection or amplification of resistance.[11] Category I can amplify resistance to rifampicin (in initial isoniazid-resistant cases) or ethambutol and pyrazinamide (in initial MDR-TB cases).[11] Category II regimen can also amplify resistance to ethambutol or streptomycin.[11]

RNTCP has ignored some important recommendations from WHO 2003 treatment guidelines for tuberculosis and it is high time to act, accept and implement the new 2010 recommendations[10] as soon as possible. The inaccuracies in the regimen should be eliminated. Evidence now shows that new cases of extra-pulmonary TB should be treated with Category-I regimen instead of Category-III regimen for better outcome.[10] WHO 2010 recommends that national TB programs need only three standard regimens:[10]

- new patient regimen: the regimen containing six months of rifampicin: 2HRZE/4HR
- retreatment regimen with first line drugs: 2HRZES/1HRZE/5HRE; and
- MDR regimen.

The estimated burden of MDR-TB under program conditions is about 1,10,132 cases according to the RNTCP 2008 data (i.e. 3% of fresh cases and 12-17% of retreatment cases are possibly multidrug resistant).[12] Adding to this pool are MDR-TB cases from treatment failures. Prevalence of MDR-TB among the failures of fresh cases ranges from 17 to 41%[13,14] and among retreatment cases ranges from 32 to 86%.[15,16] Considering that more than 50% of the new cases are not registered in RNTCP, and evaluating TB treatment practices in the private sector, the number of MDR estimates is likely to be considerably high. RNTCP DOTS-plus strategy has initiated only around 1600 MDR-TB patients on category-IV treatment. Hence there is a lot of burden to catch up. There needs to be a scaling up of the DOTS-Plus program to include all the MDR-TB patients who come to RNTCP. Given the availability of funding from international financial mechanisms, lack of resources for MDR treatment is no longer an acceptable rationale for providing a retreatment regimen of first-line drugs (the “Category II regimen”) to patients with a high likelihood of MDR.[10]

There is an urgent need for strengthening reference laboratories and laboratory network equipped with newer and rapid techniques for diagnosis of TB and drug resistance available to all the patients who present to RNTCP. Specimens for culture and drug susceptibility testing (DST) should be obtained from all previously treated TB patients at or before the start of treatment. DST should be performed for at least isoniazid and rifampicin.[10] In new patients, if the specimen obtained at the end of the intensive phase (month 2) is smear-positive, sputum smear microscopy should be obtained at the end of the third month.[10] In new patients, if the specimen obtained at the end of month 3 is smear-positive, sputum culture and drug susceptibility testing (DST) should be performed.[10] TB patients whose treatment has failed or other patient groups with high likelihood of multidrug-resistant TB (MDR-TB) should be started on an empirical MDR regimen.[17]

Relapses are not accounted for success of regimen under RNTCP and if, disease recurrence is substantial, current end of treatment targets may be too low to bring about the expected declines in incidence.[18] The program should have a provision for a minimum follow up of one year after completion of treatment as 86% of pulmonary relapse occur in first 12 months.[19]

More than 1.3 lakh TB patients were tested for HIV and more than 20,000 (hardly 2.2% of the 0.9 million total HIV-TB pool) patients are detected to be TB-HIV co-infected.[1] HIV testing for patients with TB should be made mandatory rather than optional. Also HIV patients should be regularly screened for TB. Daily dosing is now recommended for new patients with pulmonary TB throughout the course of therapy, especially for people with HIV co-infection.[20]

A public health program must not discriminate against patients by refusing care; it is not justifiable to refuse available treatment to patients in a control program. No patient should be denied treatment under RNTCP. But forced by the circumstances, the implementation of the DOTS strategy is often success driven.[21] It is even claimed by some researchers that DOTS programs rejected patients who were unlikely to adhere to treatment.[21] A New Delhi based research revealed that 37% and 49% of eligible patients received short-course treatment in two clinics, respectively. An obsession with a target cure rate can result in all other objectives being ignored.[22] DOTS came to emphasize clinical and curative aspects and surveillance, but neglected some core public health functions that are important in any setting, and more so in pluralistic health care systems.[22]

Despite these limitations, the trends in prevalence of culture positive and smear positive TB cases in South India (monitored by the Tuberculosis Research Center, Chennai) show that the decline is very rapid during the RNTCP era when compared to the pre-RNTCP era. In India, already 47% reduction has been achieved in the tuberculosis prevalence rate and 33% reduction in mortality rate in the RNTCP era. Hence, RNTCP continues to be the national and local action and commitment that determines the degree of success in tuberculosis program.

But it is high time to act to strengthen the regimens, actively involve the private sector and medical colleges and strengthen the infrastructure for DST with availability of appropriate treatment for each and every patient suffering from tuberculosis and knocking the door of RNTCP.
